# 3D Simulation-Based Acoustic Wave Resonator Analysis and Validation Using Novel Finite Element Method Software

**DOI:** 10.3390/s21082715

**Published:** 2021-04-12

**Authors:** Ruth Yadira Vidana Morales, Susana Ortega Cisneros, Jose Rodrigo Camacho Perez, Federico Sandoval Ibarra, Ricardo Casas Carrillo

**Affiliations:** 1 CINVESTAV-IPN Unidad Guadalajara, Av. del Bosque 1145, El Bajío, Zapopan 45017, Mexico; ruth.y.vidana.morales@intel.com (R.Y.V.M.); federico.sandoval@cinvestav.mx (F.S.I.); 2 Intel Corporation, Av. del Bosque 1001, El Bajío, Zapopan 45017, Mexico; rodrigo.camacho@intel.com (J.R.C.P.); ricardo.casas.carrillo@intel.com (R.C.C.)

**Keywords:** acoustic wave resonator, 3D simulation, finite element method, design techniques, cloud computing, onscale, simulation validation

## Abstract

This work illustrates the analysis of Film Bulk Acoustic Resonators (FBAR) using 3D Finite Element (FEM) simulations with the software OnScale in order to predict and improve resonator performance and quality before manufacturing. This kind of analysis minimizes manufacturing cycles by reducing design time with 3D simulations running on High-Performance Computing (HPC) cloud services. It also enables the identification of manufacturing effects on device performance. The simulation results are compared and validated with a manufactured FBAR device, previously reported, to further highlight the usefulness and advantages of the 3D simulations-based design process. In the 3D simulation results, some analysis challenges, like boundary condition definitions, mesh tuning, loss source tracing, and device quality estimations, were studied. Hence, it is possible to highlight that modern FEM solvers, like OnScale enable unprecedented FBAR analysis and design optimization.

## 1. Introduction

Acoustic Wave Resonators (AWR) have been studied starting around 50 years ago. These devices promise to improve communication platforms, sensing devices, and many other systems. AWRs are currently used in a variety of applications as Radio Frequency (RF) filters [1], gravimetric and pressure sensors [2], oscillators [3], and wearable biosensors [4,5,6], among others.

The main types of AWRs are Surface Acoustic Wave (SAW) and Bulk Acoustic Wave (BAW) resonators. They differ from each other depending on the type of waves that support resonance and its propagation from the source through or over the material [7]. BAW devices are further divided into two types, Solidly Mounted Resonator (SMR) and Film Bulk Acoustic Resonator (FBAR) [8], where the energy confinement method in the active area of the resonator is its main difference. SMR reflects the energy through a multi-layer mirror under the bottom electrode, while FBAR trap it within an air or vacuum cavity [9]. Since SMR and FBAR provide temperature stability, power handling capability, and high Q’s and coupling coefficients, efforts are currently focused on the development of novel resonator designs that take advantage of BAW characteristics and modern fabrication process technology. The coupling coefficient and quality factor are important parameters for the resonator performance to improve their design for a target application. The advent of powerful Finite Element Method (FEM) solvers capable of handling the amount of elements typically required by AWRs (in the multi-million range) opens up new possibilities to further advance the applications of AWRs. OnScale is one of such FEM solvers. It solves FEM equations in the time domain and transforms the results to frequency domain [10]. It also provides access to a Cloud High-Performance Computer (HPC) service that helps to eliminate hardware restrictions for problems with number of elements in the order of millions. Importantly, this software enables the direct analysis of acoustical and structural losses. Dielectric losses can be analyzed by post-processing the FEM solution using an additional software, like MATLAB.

The main contribution of this paper is the illustration of the FEM analysis process applied to a realistic FBAR design previously reported in Reference [11]. The analysis of this device and the comparison of simulated and measurement data results in key insights about the loss mechanisms of the device. The analysis presented in this paper highlights the importance of the availability of powerful FEM solvers and hopefully motivates the industry to further advance the state of the art of FEM solvers for AWR device design.

This work is organized as follows: Section 2 briefly presents the fundamentals of FBARs. Section 3 presents an overview of the current 2D simulations process for FBARs. Section 4 describes the 3D simulation process using OnScale. Section 5 describes the application of the simulation process to an FBAR example previously reported in the literature [11]. A discussion about the general findings of this simulation example is presented in Section 6. Finally, some conclusions are stated.

## 2. Film Bulk Acoustic Resonator Basics

### 2.1. Acoustic Wave Resonators

According to the first law of thermodynamics, energy cannot be created or destroyed, but it can be transformed from one form to another. Using piezoelectric materials, mechanical energy can be transformed into electrical (direct effect), or vice versa (inverse effect) [12]. Ideally, energy conversion must be perfect, and 100% of the electrical energy should be transformed into mechanical, or vice versa. In practice, there is a portion of the energy converted into heat, mechanical damping, or deformation.

The design of an AWR define its type of resonator. Basically, there are two general types: Bulk Acoustic Wave (BAW) and Surface Acoustic Wave (SAW) resonators. In general, the resonance frequency is directly proportional to the wavelength (λ) [13]. For acoustic resonators, physical dimensions define the wavelength of the device. For instance, in BAW resonator, piezoelectric layer thickness is λ/2, while, in SAW resonator, λ/2 is equal to the sum of one finger width plus the distance to the next finger. For this reason, for high frequency applications, SAW resonators are limited by current lithography capabilities, while BAWs are limited by deposition techniques and, consequently, by the resolution of the fabrication equipment available.

### 2.2. FBAR Design Considerations

For an FBAR design, a cavity under the device to confine the energy is required. The fabrication of this cavity presents technical challenges. For instance, the process needs to deal with high temperatures and minimize the roughness of the deposited layer or its piezoelectric properties that ultimately limit the resonator performance.

Therefore, a design of experiments study is required to determine the manufacturing conditions that result in the best resonator performance. Resonator performance is limited by material and geometric factors, among them the quality of the piezoelectric and metal layers, as well as the geometric fidelity of the cavity, anchors, electrodes, and the rest of the structure.

### 2.3. Performance Metrics

Useful comparison of resonator devices can be done by comparing performance metrics, like quality, coupling coefficient, and the Figure of Merit (FoM).

**Figure 1:**
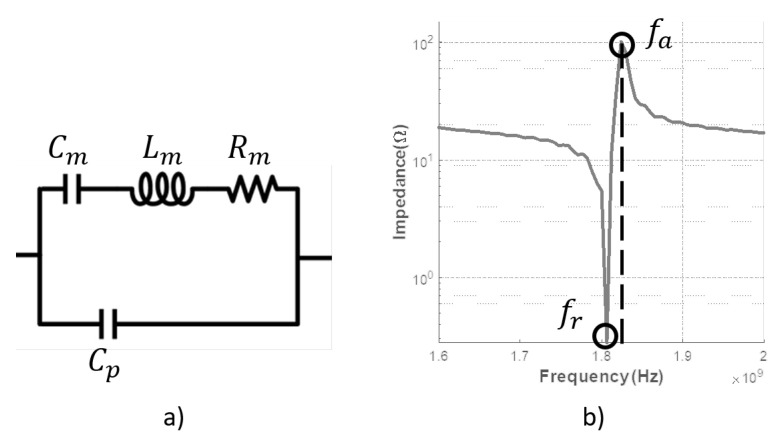
(**a**) Butterworth Van-Dyke (BVD) circuit model; (**b**) $f_{r}$ and $f_{a}$ in an impedance plot.

These quantities can be derived from the Butterworth Van-Dyke (BVD) equivalent circuit models [14,15]. The BVD circuit model is shown in Figure 1a. The value of the circuit elements can be derived from the simulated or measured impedance response of the design by solving the following expressions for the resonance $f_{r}$ and anti-resonance $f_{a}$ frequencies [16,17]:

(1) $$f_{r}=\frac{1}{\sqrt{L_{m}C_{m}}},$$

(2) $$f_{a}=\left(\frac{1}{L_{m}C_{m}}\right)\left(\frac{1+C_{m}}{2C_{p}}\right)=f_{s}\left(\frac{1+C_{m}}{2C_{p}}\right),$$

where $L_{m}$ is the motional inductor, $C_{m}$ the motional capacitor, $R_{m}$ the motional resistive loss term, and $C_{p}$ the plate capacitor of the BVD model. The corresponding resonance $f_{r}$ and anti-resonance $f_{a}$ frequencies can be read directly from the impedance response of the resonator as illustrated in Figure 1b.

The resonator Quality Factor **$Q_{f}$** represents the ratio of stored energy and energy lost on different loss mechanisms. It can be computed from the BVD circuit elements as (3):

(3) $$Q_{f}=\frac{2\pi f_{r}L_{m}}{R_{m}}.$$

In turn, the Coupling coefficient $k_{eff}^{2}$ (that shows how well the energy is converted from one form to another) is calculated using Equation (4):

(4) $$k_{eff}^{2}=\frac{\left(\frac{\pi }{2}\right)\left(\frac{f_{r}}{f_{a}}\right)}{tan\left(\left(\frac{\pi }{2}\right)\left(\frac{f_{r}}{f_{a}}\right)\right)}\approx {\left(\frac{\pi }{2}\right)}^{2}\left(\frac{f_{a}-f_{r}}{f_{a}}\right).$$

Finally, the Figure of Merit is a useful quantity for comparisons that captures both the quality and coupling coefficient, from Equations (3) and (4), as shown below in Equation (5):

(5) $$FoM=k_{eff}^{2}Q_{f}.$$

Multiple techniques can be implemented to extract BVD models based on FEM analysis as presented in Reference [18].This parameter extraction process is performed using optimization algorithms programmed in MATLAB to tune the BVD model according to measured or simulated responses.

## 3. 2D Simulations-Based Design Methodology for FBAR

Two design considerations are critical to manufacturing a resonator: the control of the manufacturing process and the knowledge of the set of parameters achieving the expected performance [19,20]. The first one allows limiting the manufacturing variations within defined ranges of variability. The second one allows focusing on the parameters minimizing unwanted effects or improving the expected performance so that the resulting device becomes as much as possible insensitive to variations.

While the design process of other microelectromechanical systems (MEMS) devices, like capacitive sensors [21,22,23], can take advantage of 3D FEM simulations to reduce the design cycle, AWRs have not benefited from 3D FEM simulations due to higher computational requirements, manufacturing complexity and lack of validation between measurements and simulation results. Three-dimensional simulations allow to develop techniques that confine energy on the resonator avoiding losses in the three axes by changing electrodes shape, adding frames, reducing anchor size, etc [24]. In addition, 3D simulations require a proper wavelength-based mesh size to capture the waves in all directions through all the material layers. In addition, for some applications, like RF filters, a broad band frequency analysis is required, as well as a small step, to capture enough details in the frequency domain [25]. To fulfill these requirements, a significant amount of RAM memory, cores availability, and powerful hardware are necessary. This is the main reason that has limited the extent and usefulness of 3D simulation analysis prior to device fabrication. Consequently, most analysis so far rely on simpler 2D simulation models.

**Figure 2:**
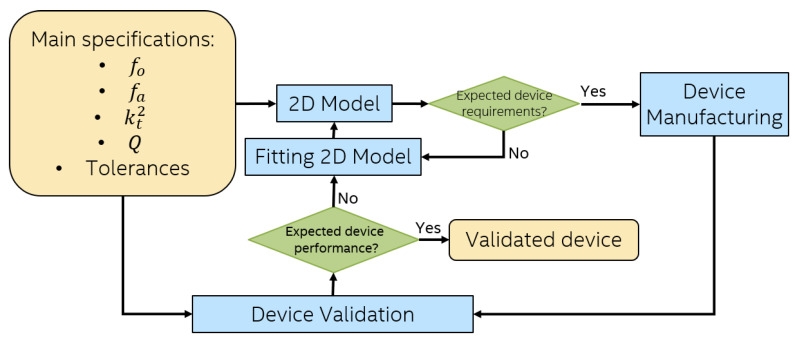
Two-dimensional simulations-based design methodology.

Current and conventional methodology for AWR design based on 2D simulation is shown in Figure 2. First, the Main specifications are provided depending on the device application and target performance. Then, a 2D model is proposed and analyzed using analytic equations and/or FEM simulations. The 2D design is adjusted/optimized until it fulfills the requirements. 2D modeling is capable to capture manufacturing tolerances. Unfortunately, the main drawback of this approach is that fabrication process defects as scalloping, slanting or undercut cannot be fully taken into account in the 2D design simulation process [26]. This is partly due to the inability of the 2D analytic formulas and FEM models to capture most realistic details of device fabrication as model variations in *x, y and z* directions generated by these defects. Consequently, in many cases, it is necessary to run several fabrication cycles to arrive at a final design in what is referred to as Device Validation in Figure 2.

## 4. 3D Simulations-Based Design Methodology for FBAR Using Onscale

In contrast to 2D simulation models, 3D simulation models capture device complexities more accurately. For example, they can capture electrode, piezoelectric layer, and cavity shapes, pads location, anchor losses, fabrication defects, etc. Nevertheless, there still exist challenges that limit the level of representation accuracy of modern 3D FEM simulation tools available today. This section outlines some of those challenges and presents a review of typical design process based on 3D simulations [27,28].

### 4.1. Main Challenges for FEM Simulations of FBAR Devices

Finite Element Method equations must be solved at each node of the complete meshed model [29]. Moreover, for proper wave phenomena representation, at least 10 or 15 mesh elements per wavelength (EPW) should be used. Hence, the larger the dimensions of the model with respect to the wavelength, the larger the number of nodes and consequently, the longer the computation time. Additionally, computing a frequency response over a broad range is required to capture multiple resonance modes and reduce spurious (not desired) resonances [30]. This results in computation time linearly increasing with the number of frequency points.

It should be noted that the size of each element and the number of elements are defined by Equations (6) and (7), respectively [13].

(6) $$i_{es}=\frac{\lambda }{EPW},$$

where $i_{es}$ is the minimum mesh element size relative to the wavelength, and

(7) $$n_{e}=\frac{v_{d}}{v_{e}},$$

where $n_{e}$ is the number of mesh elements, $v_{d}$ the device volume, and $v_{e}$ the element volume. Note, for example, that, in a case where the device volume is 814,290μm${}^{3}$, and the wavelength λ=3μm, $i_{es}$ will be 0.2μm, and $n_{e}=101,786,250$. Solving a problem of this size is a challenge even for today’s computing capabilities. Even if this example could be reduced to only 25% of its original size by applying symmetry in both X and Y axis, a swept frequency analysis of a model with 25,446,563 mesh elements is challenging and time-consuming with common software and hardware available. The solution becomes feasible only for solvers that implement efficient parallel algorithms that execute in powerful High Performance Computing (HPC) servers or farms. From this, it is evident that 3D simulation of acoustic wave resonators working at high frequencies (small wavelengths) remains a challenging computational task.

Facing these challenges, OnScale’s solvers were designed with Message Passing Interface (MPI) routines in mind, achieving unparalleled acceleration on Cloud HPC, and time domain computation to be post-processed using techniques as Fourier transform. In addition, the software performs temporal sub-sampling that can be carried out without causing aliasing of the data (fulfilling the Nyquist frequency), and then, the Fourier updates are not calculated at each time-step. All these capabilities allow to reduce the computational overhead [10].

The commercial software OnScale that implements a parallel and efficient FEM algorithm is used in this work. It is capable of solving Multi-Billion element models requiring about 1GB of RAM per 10–15 billion elements. Furthermore, the availability of an HPC cloud service from the same company allows running simulations in only few hours, and, as a consequence, it enabled the studies presented in this paper.

### 4.2. Device Design Methodology Supported by 3D Simulations

**Figure 3:**
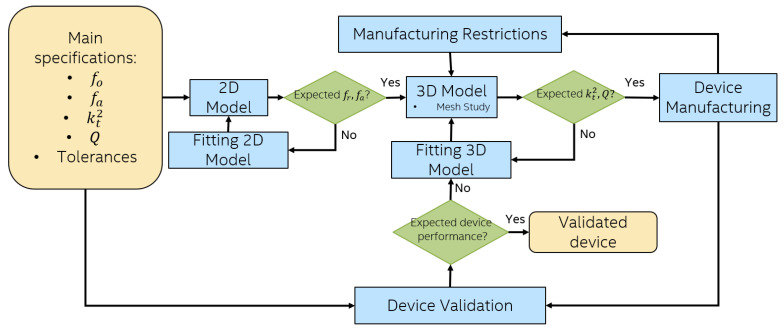
Three-dimensional simulations-based design methodology.

The methodology shown in Figure 3 can take advantage of OnScale and its capability to run complex 3D simulations at the cloud. In this scenario, complex 3D simulations become feasible. The method then allows not only to improve the design process but also to generate suitable results to reduce fabrication cycles and to take into account the fabrication restrictions on the 3D simulation.

The methodology consists of the following steps, as shown in Figure 3:

1. Main Specifications: First, the design specifications must be clearly defined. It is suitable to define criteria that can be modified to make decisions in the next steps of this methodology, as well as the tolerable range for each parameter in the design (i.e., resonance and anti-resonance frequencies, insertion loss, bandwidth, required area, etc.)
2. 2D model: Two-dimensional modeling allows to carry out a harmonic analysis in order to know the main resonance modes. In this stage, the materials of the device, their thickness and their boundary conditions are defined. The main criterion to go to the next step is the match between the resonance and anti-resonance frequencies of the model with the specifications defined in Step 1.
3. 3D model: The input for 3D modeling will be the configuration of materials and thicknesses established in Step 2: however, in the 3D simulation, the effects of the density of the materials, the shape of the electrodes, the active area, and the effect of anchors, among others, will be observed. It is very important to consider an optimized mesh resulting of the mesh study. Boundary conditions play a very important role in the simulation; for this reason, they must be well defined and located. The main criteria to go to Step 4 is the value of the mechanical coupling and the quality factor of the device. Their values will be defined by the analysis of the effects of geometry, material properties, and boundary conditions. The values of these parameters should be correlated with the tolerance ranges defined in Step 1.
4. Device Manufacturing The manufacture of the device will be carried out when a functional design is obtained in 3D simulations as expected. This is where a feedback between device manufacturing constraints and 3D modeling should be done to consider any possible effect of the manufacturing constraints. At this stage, it is convenient to define the feasibility of the prototype designed in 3D and simulate as many times as necessary the thicknesses achievable by the available deposition equipment resolution and all the modifications that the manufacturing tolerances impose on the design.
5. Device Validation: After the manufacturing process, the feedback given to the 3D simulation is very important, correcting the dielectric, acoustic or structural losses necessary so that the response in the simulation is as close as possible to the measurement of the device. In this way, the effects of the process are captured in the 3D modeling. This will allow the following cycles to consider these effects so that the designer can apply design techniques to reduce losses or suppress spurious resonances. The validation of the model will be carried out by comparing the specifications given in the first step with the result of the manufactured device. If the response of the device is far from what is expected, the cycle repeats.

**Figure 4:**
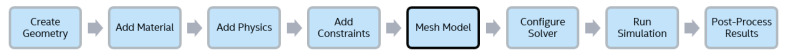
Finite Element Method (FEM) simulation process path.

Moreover, Figure 4 shows additional details about the steps taken to solve problems using 2D or 3D Finite Element solvers. In this process, a set of configurations must be completed in order to properly replicate the device conditions as material properties, single physics or multi-physics interaction, boundary conditions, proper solver, and mesh size definition. In this process, the mesh study represents a challenge for designers, since an incorrect configuration can generate unreliable data.

## 5. Study Case

The FBAR resonator presented in Reference [11] was chosen as an example to show FEM simulation capabilities and advantages of the discussed methodology. The Fabrication process, device desired thicknesses, real dimensions and proper measurements of the final device are presented in Reference [11]. This information enables us to simulate and demonstrate one of the use cases of the discussed methodology. Yet, in the present paper, the resonator is not fabricated again; hence, not all the steps of the methodology discussed above are followed. This analysis focuses on mesh tuning and capturing the manufacturing effects in order to have a 3D model that helps to optimize the device.

**Figure 5:**
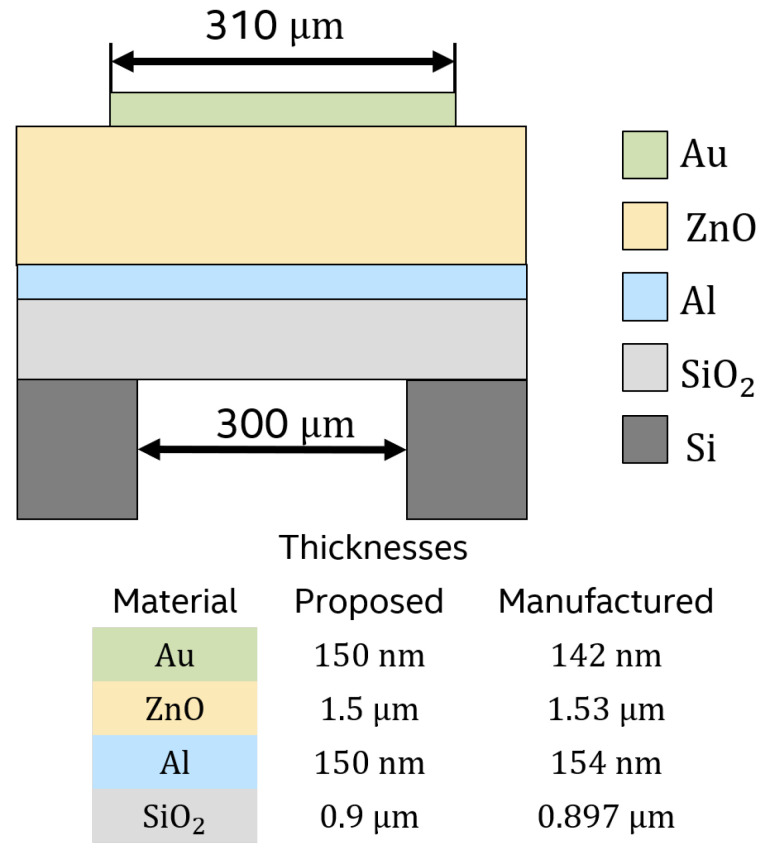
Differences between proposed and manufactured geometry.

Figure 5 shows the dimensions of the proposed and fabricated design by the authors [11]. In particular, the thickness difference is of the order of 2.66% and 5.33% for the Al and Au layers, respectively.

**Figure 6:**
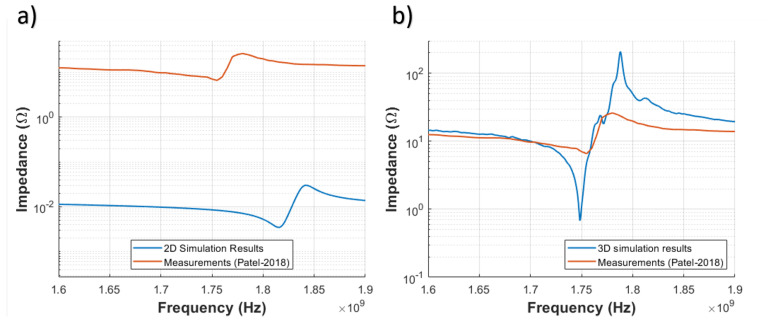
Comparison between measurements, (**a**) 2D simulation and (**b**) 3D simulation results (no losses included).

Two-dimensional simulations of manufactured were run in OnScale. Results presented in Figure 6a compare simulated and measured impedance magnitude. It is clear that both signals are different in resonance and anti-resonance frequency, and they also show different impedance magnitudes. In contrast, 3D simulation results compared with measurements from Reference [11] in Figure 6b present a similar value in resonance and anti-resonance frequency, but amplitude is completely different because the simulation represent an ideal model without losses.

### 5.1. Geometry Definition: Materials, Physics and Constraints

The 3D model was reduced to a 25% of the FBAR physical size, by applying symmetry conditions to the $x_{max}$ and $y_{max}$ planes. A mesh study was performed to discard variations due to inadequate discretization of the geometry. The target of the mesh analysis was to reproduce as close as possible the measured performance as reported in Reference [11]. Material properties for Al, Au, and ZnO were given as default from OnScale materials database since the paper chosen does not report any study related to their materials. In addition, the impedance boundary condition was configured to be automatically computed by the software for $x_{min}$ and $y_{min}$ planes. In contrast, a fixed boundary condition for $z_{min}$ plane was used and a free boundary condition for $z_{max}$ plane was set.

### 5.2. Mesh Study

Although the simulation could be run in the cloud with less restrictions of the available hardware, it is well known that saving computing resources is essential. For this reason, most designs will require a mesh study that will minimize the amount of computing resources needed to solve the model.

Moreover, meshing the model is a complex task for a 3D simulation. OnScale offers a solution for this problem. As default, it meshes the model with a uniformly distributed squared grid. While, for other simulators, the mesh is adapted to the geometry, in OnScale, the model is adapted to the mesh defined. Thus, the importance of defining properly the *"keypoints"* (forced mesh lines that help to define the geometry layers or boundaries). There are multiple options to configure the mesh in OnScale. For example, this can be done by defining the elements per wavelength (EPW) value, the minimum mesh size or the number of elements per axis, per layer, or per section.

**Figure 7:**
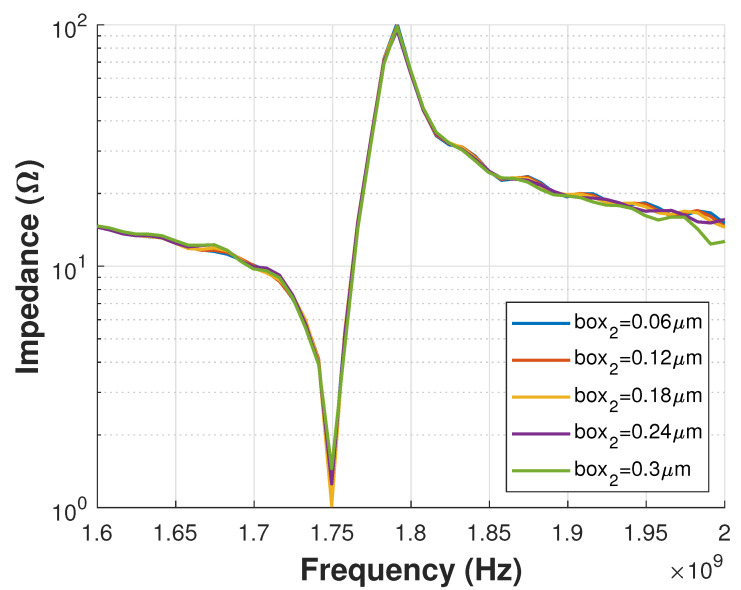
Mesh Study for $box_{2}$ that represents the mesh size in X direction.

**Figure 8:**
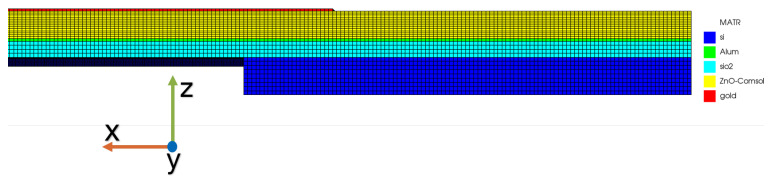
Zoom applied to the mesh definition for the 3D model.

Mesh size can be analyzed one axis at a time (x,y,z). An example of mesh size study is presented in Figure 7, where different values for mesh size $box_{2}$ over X direction were tested. As seen in the plot, the Impedance response does not show important variations, indicating that a mesh size as large as 0.3 μm can be used to reduce computational resources. Figure 8 shows a zoom view of the 3D model mesh definition. This figure shows the different mesh sizes in the Z direction for each material layer after the mesh study.

### 5.3. Losses Analysis

It is well known that different kind of losses affect the FBAR performance, and they can be analyzed in FEM simulators. The following energy balance equation can be found in Reference [10]:

(8) $$E_{IN}=E_{Elastic}+E_{kinetic}+E_{dielectric}+E_{mechanicalloss}+E_{mechanicalboundaryflow},$$

where dielectric energy ($E_{dielectric}$) is not modeled in OnScale, but the other type of energies, as elastic ($E_{Elastic}$), kinetic ($E_{kinetic}$), and mechanical ($E_{mechanicalboundaryflow}$ and $E_{mechanicalloss}$), can be simulated. Based in this equation, different losses can be considered in the OnScale simulations as acoustic (structural), anchor (boundary), viscoelastic, and electric. However, OnScale provides enough information through output files that can be post-processed in MATLAB to analyze Dielectric losses. For this example, Dielectric losses did not show a significant impact on simulation results vs measurements.

However, viscoelastic losses did show some impact. This prompted a more careful analysis of this type of loss. For the analysis of viscoelastic losses the viscoelastic constant available from measured data may not be adequate because it is obtained from measurements of macroscopic phenomena [15]. In addition, the properties of viscoelastic materials depend on many parameters, like density or stiffness [15].

In Onscale, there are multiple mathematical models available to simulate viscoelastic effects: Newtonian Viscosity (nwtn), Stiffness Proportional Damping (sdmp), Rayleigh Damping (rdmp), Viscous Damping (vdmp), and Mass Proportional Damping (mdmp).

Damping estimations can be done by describing the wave amplitude or energy and whether the loss is per cycle or per unit distance: Attenuation coeficient **α**, dB loss **dB**, Quality factor **$Q_{f}$**, Log decrements, or Fraction of critical damping ***D***.

The Rayleigh Damping (rdmp) model was chosen to develop the simulation tests presented in this work. It combines mdmp and sdmp.

The Quality factor described in Equation (9), was chosen as the parameter to be controlled in the simulation configuration to vary the viscoelasticity of materials:

(9) $$Q_{m}=\frac{\omega }{2c\alpha },$$

where $Q_{m}$ is the quality factor of the material, ω is the frequency of interest, *c* is the phase velocity, and α is the attenuation coefficient [31]. Mathematical Raleygh Model for a p-wave (*p*) and a shear wave (*s*), respectively, are shown in Equations (10)–(12),

(10) $$k_{p}=\sqrt[{}]{\frac{\rho \omega +i\gamma \omega }{B-i\omega \beta }},k_{s}=\sqrt[{}]{\frac{\rho \omega^{2}+i\gamma \omega }{G-i\omega \beta }},$$

where $k_{p,s}$ is the complex wave number for a shear or p-wave, ρ is the density, γ is the damping coefficient, B=k+4/3G is the constrained modulus, and β=k+4/3μ is the constrained viscosity.

(11) $$\alpha_{p}=Im\left[k_{p}\right],\alpha_{s}=Im\left[k_{s}\right],$$

where $\alpha_{p,s}$ is the attenuation coefficient.

(12) $$c_{p}=\frac{\omega }{Re[k_{p}]},c_{s}=\frac{\omega }{Re[k_{s}]},$$

where $c_{p,s}$ is phase velocity.

**Figure 9:**
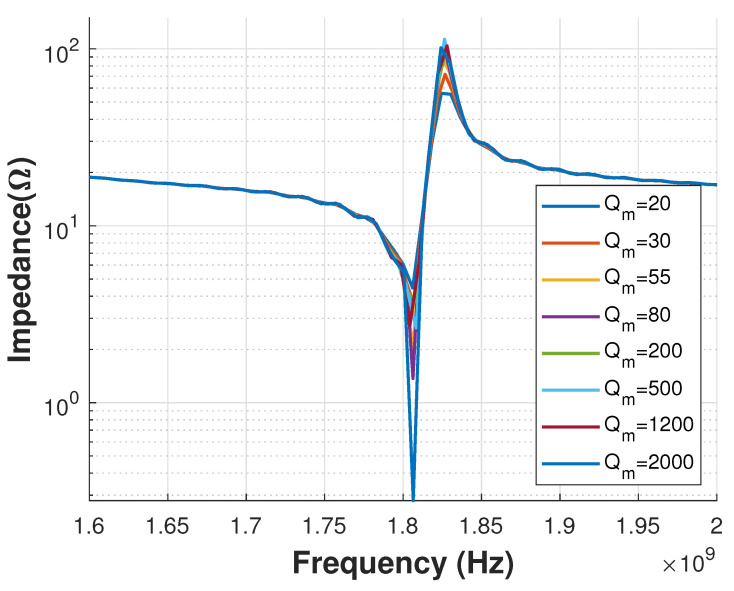
Viscous loss constant variations for bottom electrode material (Al).

The first set of simulations to analyze viscoelastic losses was focused on a sweep study of the $Q_{m}$ constant of the bottom electrode material (Al). As can be seen in Figure 9, the response was in fact affected when the losses increased. The density ρ for Al was $2690\frac{kg}{m^{3}}$, while for Au was configured as $19,700\frac{kg}{m^{3}}$, as shown in Equations (9)–(12).

**Figure 10:**
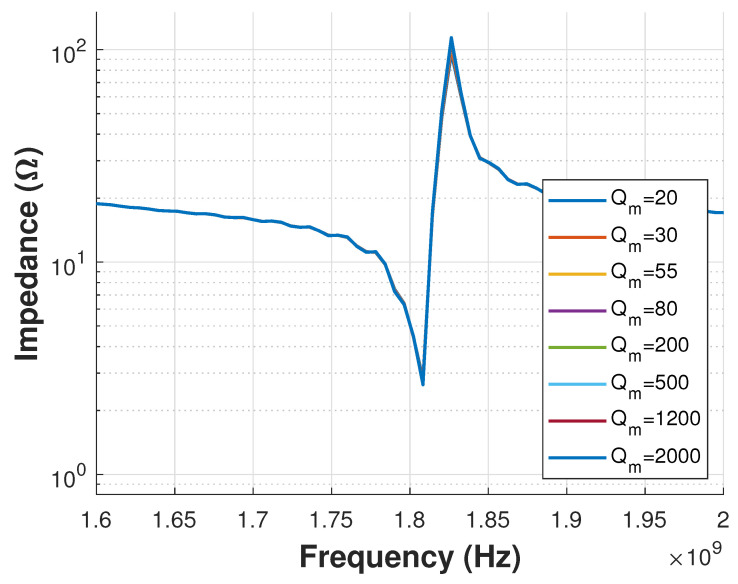
Viscous loss constant variations for top electrode material (Au).

The second set of simulations to analyze viscoelastic losses was focused on a sweep study of the $Q_{m}$ constant of the top electrode material (Au). In this case, Figure 10 shows that the $Q_{m}$ shows lower impacts than that of the bottom electrode case.

**Figure 11:**
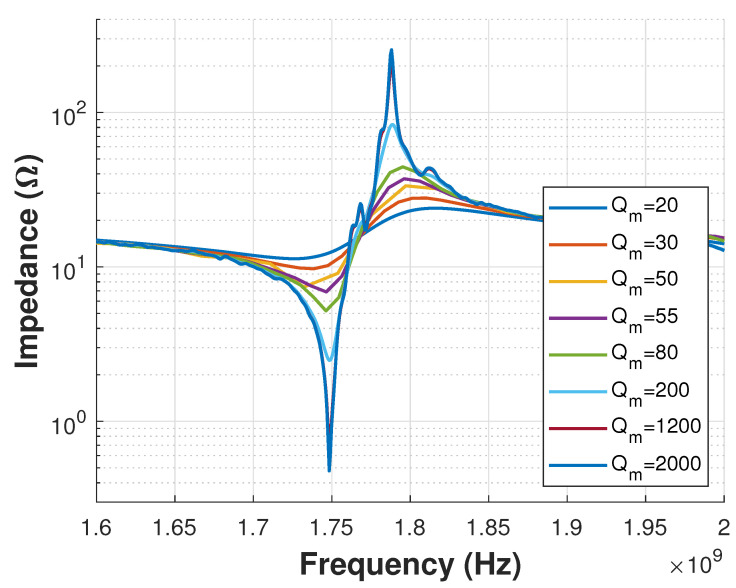
Viscous loss constant variations for piezoelectric layer (ZnO).

For the third case, the sweep study was done for the $Q_{m}$ constant of the piezoelectric layer (ZnO). This was also motivated by the reports of the effects on device quality [32]. As reported in Reference [11] for this fabricated FBAR, ZnO presents non-negligible roughness ±30 nm which can be one of the reasons of the output attenuation. Figure 11 shows that the Impedance response is clearly decreased as the loss increases.

To summarize the above analysis, the Quality Factor $Q_{f}$ of the device, its Coupling Coefficient $k_{eff}^{2}$, and its Figure of Merit FoM were computed from Equations (3)–(5). This was done for the different values of the Material Quality Factor $Q_{m}$ analyzed. Table 1 shows that FoM increases with $Q_{m}$ as expected. Yet, the $Q_{m}$ value of the deposited material is usually not known exactly beforehand due to the above mentioned limitation of the measurement method.

The piezoelectric material deposition process is a complex task since it should have crystalline characteristics, its surface also requires to be as regular as possible and its properties should be preserved as much as equipment and environment conditions allow. A complete research study of piezoelectric characteristics of ZnO films grown by RF magnetron sputtering can be found in Reference [33], experimental results demonstrate that the higher the RF power, the rougher the surface will be.

Although the manufacturing effects can not be directly configured in 3D simulations, this study shows the possibility to predict their impacts through the viscoelasticity variable configurations.

### 5.4. Comparison Results

**Figure 12:**
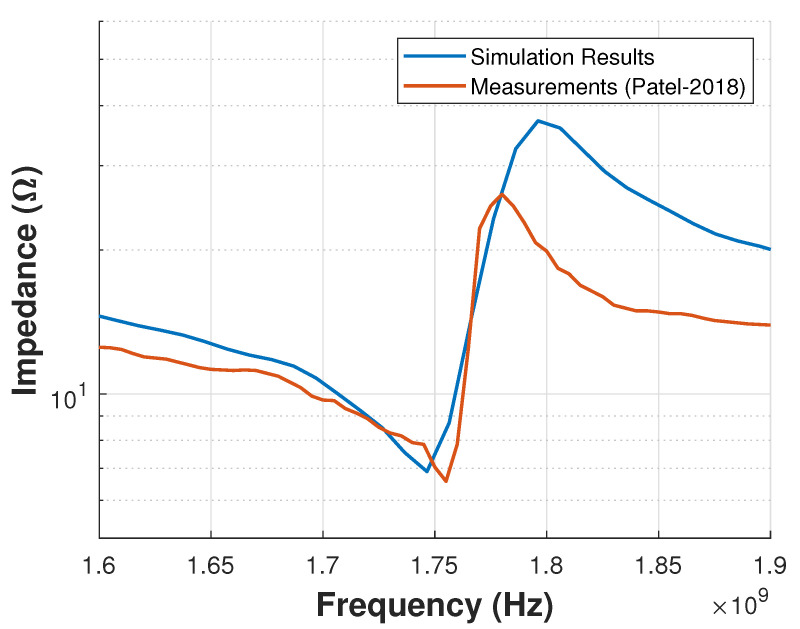
Comparison between simulated and measured impedance (magnitude).

**Figure 13:**
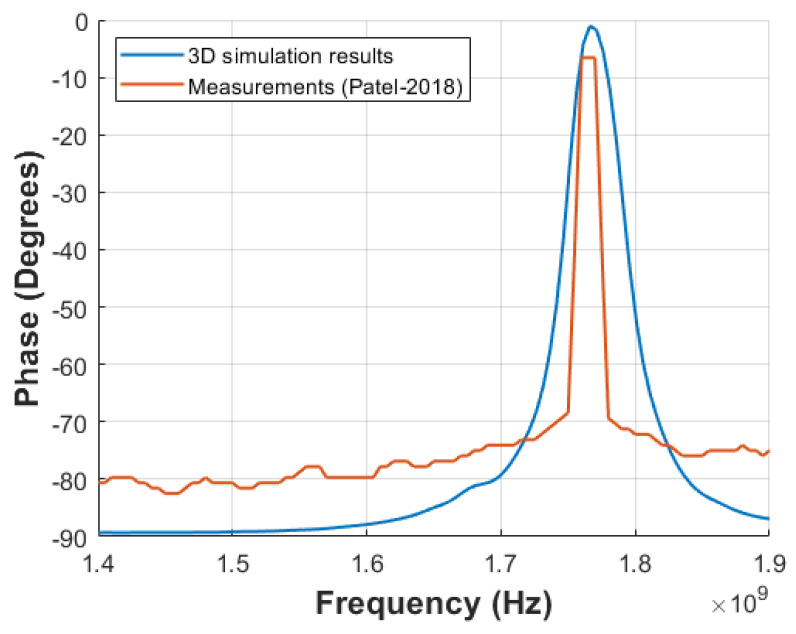
Comparison between simulated and measured impedance (phase).

To validate the proposed methodology, the value of $Q_{m}$ that results in an impedance response that best approximates the measured response is selected. Figure 12 and Figure 13 show the impedance response of the simulated device with $Q_{m}=55$ and the measured response reported in Reference [11].

A methodology for extraction of Butterworth-Van Dyke model (BVD) parameters of FBAR was programmed in MATLAB. The FBAR model frequency characteristic is obtained through FEM simulations or measurements. Then, the BVD model parameters are calculated and further optimized to fit the frequency response to those from FEM analysis and measured signal.

The circuit parameters of both BVD models are extracted from the impedance response derived from simulations and measurements, respectively, quality factor and figure of merit was calculated using (3)–(5).

Hence, two BVD models were developed corresponding with the simulations of this paper and the measurements of Reference [11]. The BVD models then enable comparable estimations of the quality and coupling coefficient.

Figure 12 and Figure 13 show the comparison of the impedance response (magnitude and phase, respectively) between the simulations results ($Q_{m}=55$) and the measurements from Reference [11]. As can be seen, there still exists a difference between the measured device behavior and simulation results. In the magnitude response, the maximum values for the measured and simulated results are 26.12 ohms and 37.2 ohms, respectively, as shown in Figure 12, whereas, in the phase response the maximum values for the measured and simulated results are -6.563 degrees and -1.019 degrees, respectively, as shown in Figure 13. Moreover, the values of FoM of the simulated and measured device deviate by less than 8%, as shown in Table 2.

## 6. Discussion

The simulation process described in Figure 3 was feasible only for MEMs devices so far. This was due to the large dimension of the devices with respect to the wavelength, as mentioned above. Fortunately, the advent of efficient FEM methods commercially available, like OnScale, such simulation process is now available for FBAR devices, as well. For instance, the analysis of the impact of the $Q_{m}$ discussed in Section 5 involved over 25 million elements and was solved in a matter of a couple of hours using OnScales’s cloud service.

The $Q_{m}$ sensitivity analysis presented in this paper highlights the importance of accurate knowledge of its value. It plays a more or less significant role depending on the type of the material (as shown in Figure 9 and Figure 10) and the frequency of interest (used to define the material properties). In the example shown in this paper, the $Q_{m}$ of the Al material at the bottom electrode impacted more significantly the performance of the device than the gold material of the top electrode. Therefore, this analysis indicates that the best option for the designer in this case is to attempt to increase the $Q_{m}$ of the Al film deposited for the bottom electrode. The stronger effect of the bottom electrode Al layer is likely due to its density being 7 times lower than the top electrode Au layer.

As mentioned above, regardless of the increasing power of solvers, like OnScale, it is still important to take advantage of all possibilities to reduce the computational complexity of the model. These include, for example, exploiting symmetry and impedance conditions, and running comprehensive mesh refinement studies prior to model optimization. OnScale also provides scripting tools, like Analyst Mode, to be able to efficiently manipulate the mesh size in all directions by layer or by section.

Additionally, it can be highlighted that the kind of analysis presented here is challenging with traditional means of calculating acoustic devices. Most of the 3D models presented in this work had more than 500 million degrees of freedom. This makes it impossible to solve in a conventional way. Three-dimensional simulations for viscoelastic losses in piezoelectric layer were run in the cloud with thousands of millions of elements. In contrast, 3D simulations for viscoelastic losses in electrodes layer were run in a local machine with hundreds million elements and results were obtained in 2–3 h. The Hardware used for local simulations was a PC with processor Intel Xeon (R)W-2135, Nvidia Quadro P1000, and 64 GB RAM memory.

Lastly, it is important to also consider at least one of the economic implications of the advent of powerful FEM solvers. The capability of solvers, like OnScale, to distribute the computation task on multiple servers on the cloud implies that companies developing AWR devices and technologies now have the option to reduce the amount of capital investment on HPC infrastructure. This investment includes not only the cost of the equipment but its maintenance, depreciation, and all the costs related to the required facilities (office space, energy, air conditioning, etc.). This may be specially important for companies, large and small, pondering the risk of AWR project development with the required significant initial investments in HPC infrastructure

In summary, the following advantages and disadvantages of the type of analysis presented in this paper may be highlighted:

Advantages:

- Mesh size optimization: Computation time can be reduced >100X when using OnScale compared with conventional software. It allows to run mesh optimization techniques for a mesh size <λ/15. OnScale provides the confidence of well meshed structures without losing wave information.
- Better capture of Manufacturing aspects: Fabrication process is a hard task when acoustic resonators are being manufactured. Fabrication defects can be included as part of the simulation in order to approach the final device before manufacturing.
- Broadband capability: Time domain solvers allow simulations to represent a broad spectrum of frequencies in the order of GHz. This allows capturing not only the main resonance frequency but a range of spurious responses, as well.
- Analysis flexibility in combination with post-processing tools: powerful tools, like OnScale, enable interfacing with general purpose numeric environments, like MATLAB. This introduces a level of flexibility to enable post-processing steps, like the ones demonstrated in this paper, related with dielectric loss analysis.
- Easier tool adoption: FEM solvers running on the cloud provide an alternative to the initial capital investment and the related costs of maintaining an HPC infrastructure.

Disadvantages:

- Learning Curve: The powerful capabilities of modern FEM solvers, like OnScale, demand learning new information about the use of the software tool. Despite the advantages of a versatile Graphical User Interface (GUI), many commands and options must be learned. Additionally, some tasks may only be configured using a lower level scripting interface mode (called Analyst mode in OnScale) that also require a learning curve.
- Frequency resolution: Common to all FEM time domain solvers, the broadband capability mentioned in the advantages introduces a trade-off in frequency resolution. Fine frequency resolution imply a larger time analysis that may potentially override the bandwidth advantage.
- Cost: Despite the advantage of leveraging the capital investments and cost of procuring and maintaining HPC infrastructure, the cost of the cloud services are still of critical consideration. Cloud services are charged by the core-hour that is a measure of the level of computing resources required. Depending on the complexity of the model, the analysis may require in the hundreds or thousands of core-hours even if following the mentioned methods to reduce computation requirements. This also increases the cost of modeling or analysis setup errors. Modeling or analysis setups that end-up in invalid simulation results also bear the consequent monetary costs of wasted cloud service time.

## 7. Conclusions

In contrast to 2D simulation models, 3D simulation models capture device complexities more accurately as shown in Figure 6. For example, they can capture design characteristics as electrode, piezoelectric layer, and cavity shapes, pads location, anchor losses, fabrication defects, etc. Nevertheless, there still exist challenges that limit the level of representation accuracy of modern 3D FEM simulation tools available today.

AWRs had not benefited from 3D simulations due to higher computational requirements, manufacturing complexity and lack of validation between measurements and 3D simulation results. The novelty of the presented work was a design methodology including a stage of OnScale 3D simulations and its validation in a fabricated model, previously reported in the literature. In the study case, the small error found in Figure 12 and Figure 13 between simulated and measured output signals (impedance magnitude and phase) showed the capability of the 3D simulations to approach a manufactured model. These obtained results highlight the importance of including 3D simulations in the proposed AWRs design methodology (illustrated in Figure 3).

The advent of powerful 3D FEM simulators, like OnScale, opens multiple possibilities to accelerate and optimize the FBAR design process. This paper illustrates the analsysis of an FBAR design based on 3D FEM simulations using OnScale. The analysis highlights the possibility of device design optimization considering the impact from fabrication process aspects, like the quality of material deposition. The analysis presented in this paper was performed using OnScale’s HPC cloud service. This enabled solution times in the order of hours instead of days. Hence, it becomes possible to run detailed studies, like a mesh study to ensure proper device numerical representation or configure the timestep to properly capture the model behavior in the steady-state.

The analysis was performed on the FBAR device reported in Reference [11]. The analysis allowed to identify the effects of the viscoelasticity losses in the impedance response. The simulation results of this paper were compared with the measured data presented in Reference [11]. The bottom electrode (Al), piezoelectric (ZnO), and top electrode (Au) layers were studied independently. It was found that material density was the parameter that increases or reduces the viscoelastic effects on device performance.

These kind of analysis also enable fabrication process feedback, since the consideration of poor quality material deposition or temperature processes effects will provide more reliability in the simulation results. Some of these effects were taken into account by configuring losses in the simulation model.

The application of this methodology can be extended to the AWR design process to obtain improved performance devices in less time and using less resources. It will also give the chance to apply optimization techniques to the device before fabrication and will accelerate the research stage on novel resonator designs that minimize losses and provide spurious resonance suppression. The proposal suggests that the results can be used in other applications of the AWR design and other areas of multiphysics systems. The analysis of FBARs with complex or unsymmetrical geometries will be studied. The extension to other types of AWRs and multiple divices effects in the same die also will be considered in a future work.

## Figures and Tables

**Table 1:** $f_{r}$, $k_{eff}^{2}$, $Q_{f}$, and FoM for different $Q_{m}$ values in piezoelectric layer.

	$Q_{m}$ = 50	$Q_{m}$ = 55	$Q_{m}$ = 80	$Q_{m}$ = 200	$Q_{m}$ = 1200	$Q_{m}$ = 2000
$f_{r}\left[GHz\right]$	1.7432	1.7464	1.7463	1.7483	1.7464	1.7464
$k_{eff}^{2}$	8.92	6.82	6.73	5.58	5.48	5.48
$Q_{f}$	28.61	39.83	53.67	143.5	541	767
FoM	2.55	2.68	3.62	8.00	29.65	42.03

**Table 2:** Quality and coupling comparison: Measurements and simulations.

	Measurements	3D Results
$f_{r}$ [GHz]	1.75	1.7464
$k_{eff}^{2}\left[\%\right]$	4.154	6.82
$Q_{f}$	59.8	39.83
FoM	2.48	2.68

## Data Availability

No new data were created or analyzed in this study. Data sharing is not applicable to this article.
